# Optimizing malignancy prediction: A comparative analysis of transfer learning techniques on EBUS images

**DOI:** 10.1016/j.clinsp.2025.100703

**Published:** 2025-06-14

**Authors:** Ali Erdem Ozcelik, Neslihan Ozcelik, Emre Bendes, Gizem Ozcibik Isik, Omer Topaloglu

**Affiliations:** a Department of Landscape Architecture, Recep Tayyip Erdogan University, Faculty of Engineering and Architecture, Turkey; b Department of Pulmonary Medicine, Recep Tayyip Erdogan University, Faculty of Medicine, Turkey; c Department of Computer Engineering, Nevşehir Haci Bektas Veli University, Faculty of Engineering-Architecture, Turkey; d Department of Thoracic Surgery, Bolu Izzet Baysal State Hospital, Turkey; e Department of Thoracic Surgery, Recep Tayyip Erdogan University, Faculty of Medicine, Turkey

**Keywords:** EBUS, Lung cancer, Machine learning, Transfer learning, VGG, ResNet, InceptionNet

## Abstract

**Background:**

Improving diagnostic accuracy in EBUS image analysis using machine learning is a current challenge. This study aimed to identify the most effective transfer learning model for predicting lymph node malignancy.

**Methods:**

EBUS images collected between 2020–2023 were retrospectively analyzed. Demographic data, sampled lymph nodes, and pathology results were retrospectively collected from the files. Eight pre-trained CNN models (VGG, ResNet, InceptionNet, Xception, MobileNet, DenseNet, NasNet, EfficientNet) were evaluated.

**Results:**

The study shows that the VGG19, EfficientNetV2L and DenseNet201 models have the highest performance in malignancy prediction, achieving areas under the curve of 0.96, 0.96 and 0.95 respectively, with consistent training and testing accuracy, indicating successful models without overfitting. In contrast, the ResNet152V2, Xception, and NasNet models show lower performance with areas under the curve of 0.88, 0.85, and 0.84 respectively, indicating overfitting due to discrepancies between training and test data. The MobileNetV2 model, with an area under the curve of 0.50, fails to discriminate between benign and malignant cases, resulting in an accuracy of only 0.51.

**Conclusions:**

The application of transfer learning to the analysis of EBUS images offers significant potential for improving diagnostic accuracy in thoracic medicine, particularly in lung cancer.

## Highlights

• EBUS enhances mediastinal lymph node evaluation with less invasiveness.
• VGG19, EfficientNet, and DenseNet achieved top malignancy prediction (AUC = 0.96).
• Model architecture plays a key role in selecting transfer learning approaches.

## Introduction

Mediastinal lymph nodes play a pivotal role in diseases such as lung cancer, tuberculosis, lymphoma, sarcoidosis, and mediastinal tumors. Accurate diagnosis and staging are essential for determining treatment strategies.^1^ Although surgical approaches remain standard, their invasiveness and need for general anesthesia have increased interest in less invasive diagnostic alternatives.

Endobronchial Ultrasound (EBUS), a minimally invasive method integrating ultrasonography with bronchoscopy, allows direct access to lesions such as enlarged lymph nodes or bronchus-adjacent tumors that classical bronchoscopy could not reach.^2^ EBUS enables real-time visualization, guided biopsy, and Doppler-based vascular distinction, significantly advancing the diagnosis and staging of mediastinal diseases.

Recent studies have shown that sonographic features from EBUS images can support diagnosis. Distinguishing malignant from benign lymph nodes is essential to avoid unnecessary biopsies. Due to the operator-dependent nature of current assessments, objective, computer-based decision support systems are needed to ensure consistent evaluations.^3^ The authors anticipate the development of computer-based decision support systems to deliver more objective results, independent of the operator.

Various machine-learning approaches have been applied to EBUS images, yet the optimal method for accurate malignancy prediction remains under investigation.^4^, ^5^, ^6^, ^7^, ^8^, ^9^

Most studies utilize publicly available datasets, with ImageNet being the predominant pre-training source due to its scale and accessibility.^10^ Increasing this rate over the next few years will be essential for improving methods and comparing performance. In most studies, ImageNet was chosen as the pre-training data source because of its size and ease of use.^10^ Among the most commonly used models are DenseNet and ResNet, the two popular Convolutional Neural Network (CNN) models.^11^^,^^12^ Rather than more complex hybrid methods (e.g., CheXNet), it has been observed that models such as DenseNet and ResNet, which can be easily trained with programs such as PyTorch or Keras, are preferred for TL, since they are pre-trained with ImageNet.^13^^,^^14^ It is seen in the literature that these methods are used in different studies.^15^

## Objective

The aim of this study is to analyze images obtained from EBUS procedures using artificial intelligence methods and compare the results with pathology outcomes. The primary aim of this study is to distinguish between benign and malignant lymph nodes, rather than differentiate among histologic subtypes of malignancy. The goal is to identify the best transfer learning method for optimizing malignancy prediction.

## Materials and methods

### Patients’ characteristics, EBUS procedure and data collection

Two bronchoscopists experienced with EBUS-TBNA performed all of the procedures. In this study, the bronchoscopists’ role was to image the LNs with EBUS and perform a biopsy. The bronchoscopists performed the imaging process following the common standards of the EBUS procedure followed by all medical experts in this field and accepted worldwide.^16^

**Fig. 1:**
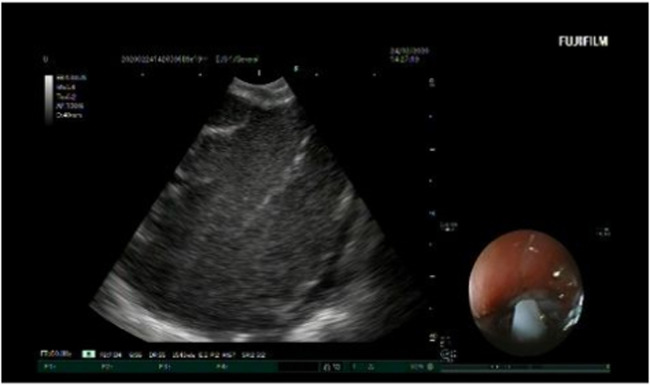
Representative image from an Endobronchial Ultrasound (EBUS) procedure showing the mediastinal Lymph Node (LN) adjacent to the airway wall. The image was captured during a real-time Transbronchial Needle Aspiration (TBNA) session.

EBUS was performed under general anesthesia. The pulse rate, respiratory rate, oxygen saturation, and blood pressure were monitored throughout the procedure. A Convex-Probe Endobronchial Ultrasound (CP-EBUS) scope (Fujifilm, Ultrasonic Processor SU-1) with a 7.5 MHz convex transducer was used. The ultrasonic processor processed B-mode and color-power Doppler images. CP-EBUS was performed orally. International Staging System guidelines for lung cancer were followed to identify the LNs. After identifying the node with EBUS, the vascular structures were detected using the Doppler mode. The images were captured during a real-time Transbronchial Needle Aspiration (TBNA) session (Fig. 1). The authors used a 22-gauge cytology needle to obtain specimens. The samples obtained from the LNs were spread on glass, and fixed with 95% alcohol and formalin. These were then sent to the Pathology Department, and the pathology results were obtained. The patient’s images were classified as malignant or benign, according to the pathology results. Based on the expert visual interpretation of pulmonologists, each image was evaluated independently by the same observer.

The data used in this study were collected from the images obtained from the EBUS procedures performed in the clinic between 2021 and 2023. Demographic data, sampled lymph nodes, and pathology results were retrospectively recorded from patient files. Histopathological evaluation of biopsy specimens obtained via EBUS-TBNA was considered the gold standard for the diagnosis of malignancy.

### Image processing and data augmentation

The dataset comprised 310 EBUS images. To increase the power of the dataset, each image was subjected to 36 rotations and 10 distortions. To avoid class imbalance or artificial bias, augmentation procedures were applied equally across benign and malignant classes using randomized parameters that preserved anatomical fidelity. The transformations included uniform rotations and mild distortions applied symmetrically across classes, minimizing the risk of bias or overfitting due to augmentation artifacts. Thus, a total of 14,570 images were acquired for the dataset. 20 % of the dataset was used for testing, while the remaining 80 % was used for training. Training and test data were selected under a random process. Diagnosis of benign and malignant conditions in the datasets occurred at comparable frequencies. Homogeneity was guaranteed in both the training and test groups. Through this approach, the precision and dependability of the artificial intelligence models were enhanced. 11,656 images were allocated as training data for each artificial intelligence model, while 2914 images were designated as test data. The development of artificial intelligence models involved the use of 8 training algorithms. The objective was to choose the optimal model by evaluating the superiority, success, and confidence levels of the artificial intelligence models in relation to one another.

### Comparison of transfer learning models

This study utilized Convolutional Neural Network (CNN) models integrated with Transfer Learning (TL). Under the TL approach, AI models retain previously learned patterns to solve new problems more efficiently and with less data.^17^ CNNs, commonly used in image processing, are deep learning algorithms designed for image classification and benefit from TL for faster and data-efficient training.^18^ CNNs, commonly used in image processing, are deep learning algorithms designed for image classification and benefit from TL for faster and data-efficient training.^19^ The models evaluated were VGG, ResNet, InceptionNet, Xception, MobileNet, DenseNet, NasNet, and EfficientNet.

Each algorithm offers distinct advantages and limitations, and these are briefly summarized below.

#### Visual geometry group (VGG)

VGG19 is a transfer learning-based convolutional network with 19 layers, including 16 convolutional and 3 fully connected layers. In this study, input and output dimensions were set as 224 × 224 × 64 and 1 × 1 × 100, respectively.^20^

#### Residual network (ResNet)

ResNet uses shortcut connections (residual blocks) to allow deeper networks while avoiding vanishing gradient issues. This structure facilitates optimization and improves accuracy in very deep models.^21^

#### Inception residual network V2 (InceptionResNetV2)

InceptionResNetV2 combines the Inception architecture’s parallel convolutions with ResNet-style residual connections to enhance performance while reducing computational load.^22^

#### Xception

Xception improves upon InceptionV3 by using depthwise separable convolutions, which reduce computational cost while maintaining accuracy.^23^

#### MobileNetV2

MobileNetV2 employs depthwise separable convolutions and skip connections to reduce model size and computational cost, making it suitable for low-resource environments.^24^

#### DenseNet201

DenseNet201 connects each layer to all subsequent layers, improving gradient flow and feature reuse while reducing parameters and overfitting risk.^25^

#### NasNet

NasNet uses Neural Architecture Search (NAS) to optimize model design based on the dataset, improving performance through adaptive block structures.^26^

#### EfficientNet

EfficientNetV2L combines neural architecture search with compound scaling to balance accuracy, training speed, and model efficiency.^27^

### AI workflow and outcome measures

**Fig. 2:**
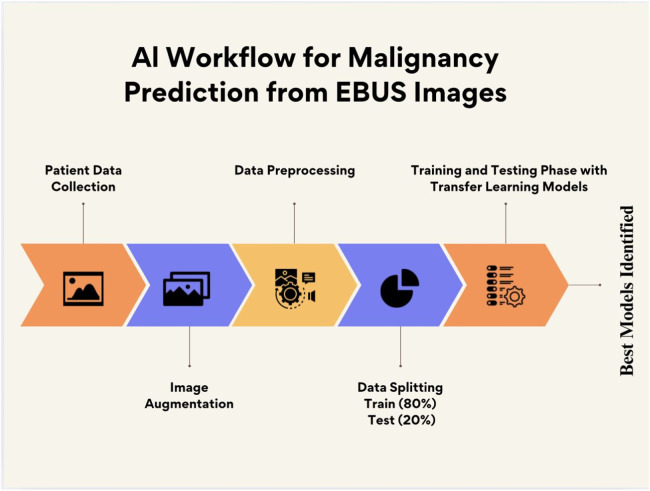
Workflow of the deep learning-based malignancy prediction process. The diagram illustrates key steps from EBUS image acquisition and augmentation, through training and testing data separation, to model training and evaluation using transfer learning methods.

Fig. 2 shows the workflow of the deep learning-based malignancy prediction process. The diagram illustrates key steps from EBUS image acquisition and augmentation, through training and testing data separation, to model training and evaluation using multiple pre-trained convolutional neural networks.

As seen in Fig. 2,the primary outcome was the diagnostic performance (AUC, accuracy) of each transfer learning model in predicting malignancy from EBUS images.

### Statistical analysis

The performance of each deep learning model was evaluated using standard classification metrics, including Area Under the Receiver Operating Characteristic Curve (AUC), accuracy, sensitivity, specificity, and loss values. Accuracy and loss values were calculated separately for both training and testing datasets to assess model generalizability and detect overfitting. The compatibility of training and test data was visually examined through accuracy and loss function curves across epochs. Comparisons between models were based primarily on their AUC values and consistency between training and test performances. All statistical analyses and model evaluations were performed using Python (version 3.11.9) with Keras and TensorFlow libraries. A p-value was not used, as the primary focus was on algorithmic performance rather than hypothesis testing.

### Ethics approval

The methodology for this study and ethical considerations for human subjects were reviewed and approved before performing the present study by the Human Research Ethics Committee of Recep Tayyip Erdogan University (Ethics approval number: 2021/59). Due to the retrospective design of this study, informed consent was not obtained from the patients.

All data used in this study were anonymized before analysis. No personally identifiable information was collected or processed. The study was conducted in accordance with the Declaration of Helsinki.

## Results

**Fig. 3:**
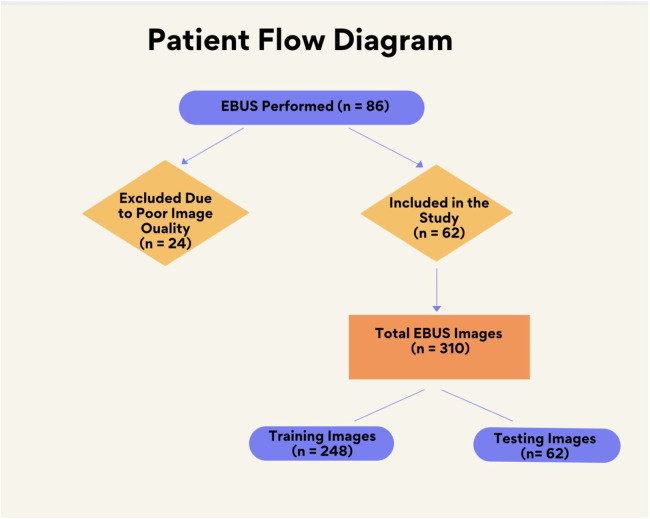
Patient flow diagram.

The dataset comprised 310 EBUS images from 62 patients (Fig. 3). To increase the power of the dataset, each image was subjected to 36 rotations and 10 distortions. Thus, a total of 14,570 images were acquired for the dataset. 20 % of the dataset was used for testing, while the remaining 80 % was used for training. Patient characteristics and data are given in Table 1.

**Table 1:** Patient characteristics and demographic data.

Characteristics	Cases
Patients, n	62
Sex (Male/Female)	44/18
Age, Median (Range), y	64 (23–84)
Diagnosis of patients, n (%)	
Lung cancer	38 (61.3)
Adenocarcinoma	30 (79)
Squamous carcinoma	6 (15.8)
Small cell lung cancer	2 (5.2)
Granulomatous inflammation	24 (38.7)
Lymph nodes, *n*	310
Long axis on EBUS, mm	31.36 ± 7.52
Short axis on EBUS, mm	14.80 ± 6.87
Station, *n* (%)	
2R, 4R, 4L	75 (24.25)
7	163 (52.5)
10R, 10L	65 (21)
11 L, 11R	7 (2.25)

**Fig. 4:**
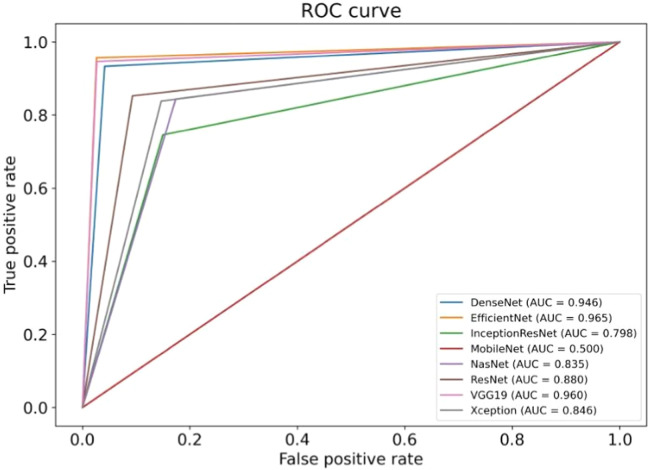
ROC curve graph of the models. The evaluation of the models' success relied on the area under the curve. ROC, Receiver Operating Characteristic; AUC, Area Under the Curve.

**Fig. 5:**
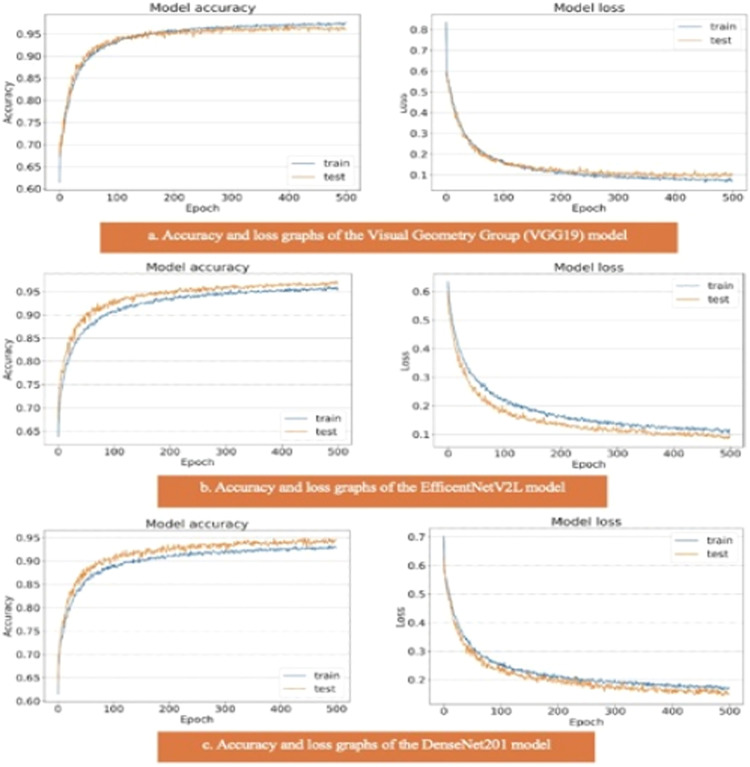
(a) Accuracy and loss graph of the Visual Geometry Group (VGG19) model. (b) Accuracy and loss graph of the EfficentNetV2L model. (c) Accuracy and loss graph of the DenseNet201 model.

The results of the artificial intelligence models are presented in Table 2. We see that the VGG19, EfficientNetV2L, and DenseNet201 models have the most successful results, with the area under the curve being 0.96, 0.96, and 0.95 (Fig. 4). The graphs (Fig. 5) display the accuracy results of the training and test data for the VGG19, EfficientNetV2L, and DenseNet201 models. The training and test model graphs increase at the same rate, while the loss function graphs decrease at the same rate as the iterations increase. The accuracy and model loss function graphs of these three models show that the training and test data are compatible, that they are successful, and that there is no overlearning. Of these three models, VGG19 has the most successful results.

**Table 2:** The table displays the test results of the models.

Models	Specificity	Sensitivity	PPV	NPV	Accuracy	AUC
VGG19	0.97	0.95	0.97	0.95	0.97	**0.96**
ResNet152V2	0.91	0.85	0.90	0.86	0.88	0.88
InceptionResNetV2	0.85	0.75	0.83	0.78	0.81	0.80
Xception	0.85	0.84	0.85	0.84	0.85	0.85
MobileNetV2	1	0	Nan	0.51	0.51	0.50
DenseNet201	0.96	0.93	0.96	0.94	0.95	**0.95**
NasNet	0.83	0.84	0.83	0.85	0.84	0.84
EfficientNetV2L	0.97	0.96	0.97	0.96	0.97	**0.96**

AUC, Area Under the Curve, PPV, Positive Predictive Value, NPV, Negative Predictive Value.

**Fig. 6:**
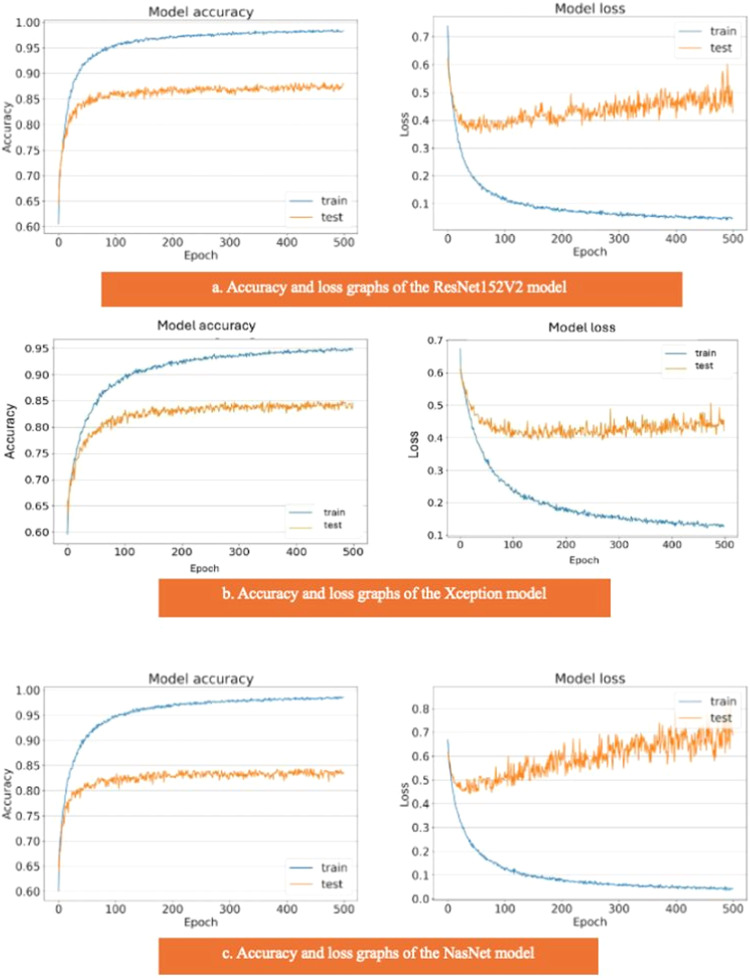
(a) Accuracy and loss graphs of the ResNet152V2 model, (b) Accuracy and loss graphs of the Xception model, (c) Accuracy and loss graphs of the NasNet model.

The ResNet152V2, Xception, and NasNet models had areas under the curves of 0.88, 0.85, and 0.84, respectively (Fig. 4). These are the graphs showing the accuracy results of the training and test data for the ResNet152V2, Xception, and NasNet models. The training and test model graphs grow at different rates, and as iterations increase, the loss function graphs become more separated (Fig. 6). The incompatibility of the training and test data graphs, as observed in the graphs of these three models, indicates that overlearning is occurring in the models, leading to unsuccessful results.

**Fig. 7:**
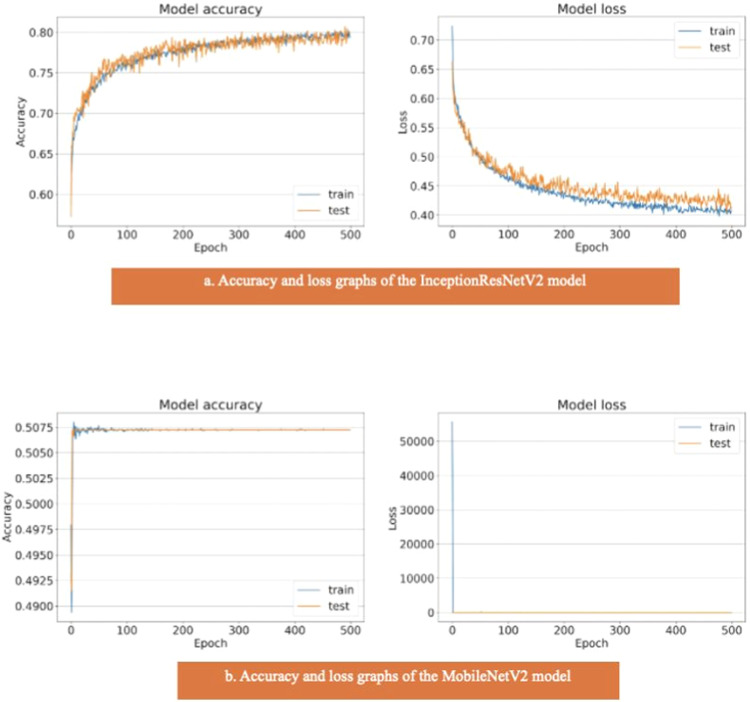
(a) Accuracy and loss graphs of the InceptionResNetV2 model, (b) Accuracy and loss graphs of the MobileNetV2 model.

The areas under the curves of the InceptionResNetV2 and MobileNetV2 models were 0.80 and 0.50, respectively (Fig. 4). The training and test model curves do not grow at the same rate for the InceptionResNetV2 model. Also, the loss function graphs grow further apart as the number of iterations increases (Fig. 7). The graphs of the training and test data in the model are incompatible. We see that the model is overlearning, which leads to unsuccessful results. When we look at the accuracy graph of the MobileNetV2 model, we see that the training and test accuracy values plateaued early and did not improve with further iterations, suggesting poor learning behavior, while the model loss function graph (Fig. 7) shows the test graph as 0. The model fails because it classifies all the images as benign, and its accuracy value is 0.51.

**Fig. 8:**
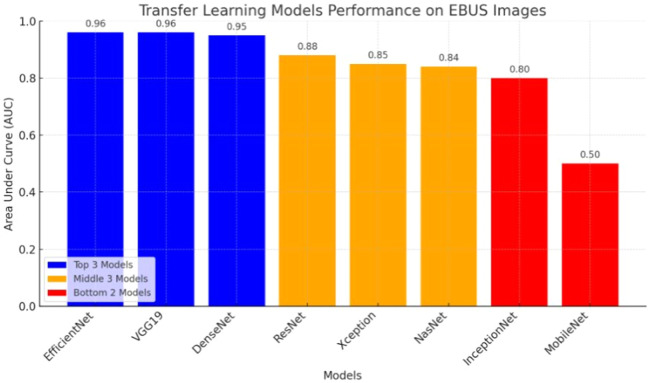
The performance comparison of transfer learning models based on their Area Under the Curve (AUC) scores.

Fig. 8 visualizes the performance comparison of transfer learning models based on their Area Under the Curve (AUC) scores. VGG19, DenseNet201, and EfficientNet demonstrated the highest performance with an AUC of 0.96, while MobileNetV2 showed the lowest performance with an AUC of 0.50.

## Discussion

This study compared eight deep transfer learning models for EBUS image classification. Among them, VGG19, EfficientNetV2L, and DenseNet201 showed the highest performance with AUC values above 0.95.

Transfer learning has emerged as a key technique in medical image analysis, enabling the development of robust models that can effectively classify and segment medical images. This approach uses pre-trained models, especially those trained on large datasets such as ImageNet, to improve performance on specific medical tasks.^28^

There have been several studies showing the effectiveness of transfer learning methods in medical ultrasound image analysis. In breast ultrasound studies, cross-domain transfer learning has shown promise. Ayana et al. demonstrated improved performance by adapting models pre-trained on natural images,^29^ while Masud et al. reported that NASNet achieved 99 % accuracy in breast cancer classification.^30^ This indicates that specific architectures can significantly impact the effectiveness of transfer learning in ultrasound diagnostics.

Similarly, Cheng and Malhi demonstrated that CNN layers can act as effective feature extractors in abdominal ultrasound, even with noisy data, using a relatively small dataset.^31^ Saha and Sheikh emphasized that differences between natural and ultrasound images may limit transferability, necessitating domain-specific adaptation.^32^ This highlights the necessity for careful selection of pre-trained models and the potential need for additional domain-specific training.

Expanding further, Wu et al. employed InceptionResNetV2 and class activation maps to visualize predictive features in thyroid ultrasound, enhancing the interpretability of deep learning models.^33^ This capability is crucial for medical professionals who require transparency in AI-driven diagnostics.

Chen et al. showed that preprocessing techniques such as total variation-based restoration, combined with GoogLeNet, improved the classification of thyroid nodules.^34^ These studies suggest that data augmentation and careful preprocessing are essential for optimizing model performance.

EBUS-TBNA is a key minimally invasive tool for evaluating mediastinal lymph nodes and has become a first-line method for lung cancer staging, with sensitivity reported up to 90 %.^35^ AI-based decision support tools can assist clinicians in planning re-biopsies or follow-up strategies when initial results are inconclusive.^35^

Recent research has focused on integrating AI with EBUS imaging. CNN-based approaches have been developed to detect and classify lymph nodes in real time, reducing operator dependence and enhancing diagnostic accuracy.^36^ In particular, Convolutional Neural Networks (CNNs) have shown promising results in reducing operator dependence and improving diagnostic accuracy.

The application of deep learning algorithms in ultrasound imaging has been demonstrated to yield high predictive values in various medical contexts, suggesting that similar methodologies could be adapted for EBUS image analysis.^37^ There are studies to compare which ML model is more effective in analyzing EBUS images. Koseoglu et al. found that fine-tuned ML applications like SVM and KNN can significantly enhance the analysis of EBUS images, improving diagnostic accuracy.^38^

Consistent with the present findings, Patel et al. achieved 80.6 % accuracy and an AUC of 0.701 using an ensemble CNN model on EBUS images. Despite high specificity (96.9 %), their model showed low sensitivity (43.2 %), underscoring the need for improved sensitivity and validation on multicenter datasets.^39^ Their results emphasize the feasibility of AI-supported diagnostic tools in real-time EBUS interpretation and support the development of ensemble models trained on diverse datasets to improve clinical applicability.

Transfer learning offers substantial potential in EBUS image analysis by enabling the extraction of complex diagnostic features. These models can enhance accuracy and reduce subjectivity, potentially matching the diagnostic utility of surgical mediastinoscopy for mediastinal and hilar lymphadenopathies.^40^

### Limitations

This single-center study may limit the generalizability of the findings. Although the models showed strong performance, the absence of external validation may affect their robustness in broader clinical settings. Furthermore, the models were not tested for identifying malignancy subtypes. Future multi-center studies with larger, diverse datasets and external validation are needed to confirm applicability.

While the dataset is not publicly available due to institutional policies, we are open to multicenter collaborations, and anonymized subsets may be shared under appropriate data-sharing agreements following ethical approval.

## Conclusions

The application of transfer learning to the analysis of Endobronchial Ultrasound (EBUS) images offers significant potential for improving diagnostic accuracy in thoracic medicine, particularly in lung cancer. By utilizing deep learning and advanced imaging methods, clinicians can improve patient outcomes through more accurate diagnoses.

### Institutional review board statement

This study was performed in line with the principles of the Declaration of Helsinki. Approval for the study was granted by the Ethics Committee of Recep Tayyip Erdogan University. The ethics committee approval number is 2021/59.

### Informed consent statement

Due to the retrospective design of the present study, informed consent was not obtained from the patients.

## Data availability statement

All data generated or analyzed during this study are included in this article. The data will be available upon reasonable request (contact persons: alierdem.ozcelik@erdogan.edu.tr and omer.topaloglu@erdogan.edu.tr).

## Authors’ contributions

All authors contributed to the study's conception and design. Material preparation, data collection, and analysis were performed by A.E.O, N.O., E.B., G.O.I. and O.T. The first draft of the manuscript was written by A.E.O., N.O., G.O.I. and O.T. All authors commented on previous versions of the manuscript. All authors have read and agreed to the published version of the manuscript.

## Funding

This research did not receive any specific grant from funding agencies in the public, commercial, or not-for-profit sectors.

## Declaration of competing interest

The authors declare that they have no known competing financial interests or personal relationships that could have appeared to influence the work reported in this paper.

## References

[1] Philip B., Jain A., Wojtowicz M., Khan I., Voller C., Patel R.S.K. (2023). Current investigative modalities for detecting and staging lung cancers: a comprehensive summary. Indian J Thorac Cardiovasc Surg.

[2] Vilmann P., Clementsen P.F., Colella S., Siemsen M., De Leyn P., Dumonceau J.M. (2015). Combined endobronchial and esophageal endosonography for the diagnosis and staging of lung cancer: european Society of Gastrointestinal Endoscopy (ESGE) Guideline, in cooperation with the European Respiratory Society (ERS) and the European Society of Thoracic Surgeons (ESTS). Endoscopy.

[3] So C., Matsumoto Y., Imabayashi T., Uchimura K., Ohe Y., Furuse H. (2023). Identifying factors causing failure of nodal staging by endobronchial ultrasound-guided transbronchial needle aspiration in non-small cell lung cancer. Transl Lung Cancer Res.

[4] Liu E., Bhutani M.S., Sun S. (2021). Artificial intelligence: the new wave of innovation in EUS. Endosc Ultrasound.

[5] Lin C.K., Wu S.H., Chua Y.W., Fan H.J., Cheng Y.C. (2025). TransEBUS: the interpretation of endobronchial ultrasound image using hybrid transformer for differentiating malignant and benign mediastinal lesions. J Formos Med Assoc.

[6] Yong S.H., Lee S.H., Oh S.I., Keum J.S., Kim K.N., Park M.S. (2022). Malignant thoracic lymph node classification with deep convolutional neural networks on real-time endobronchial ultrasound (EBUS) images. Transl Lung Cancer Res.

[7] Patel Y.S., Gatti A.A., Farrokhyar F., Xie F., Hanna W.C. (2024). Artificial intelligence algorithm can predict lymph node malignancy from EBUS-TBNA images for NSCLC. Respiration.

[8] Ito Y., Nakajima T., Inage T., Otsuka T., Sata Y., Tanaka K. (2022). Prediction of nodal metastasis in lung cancer using deep learning of endobronchial ultrasound images. Cancers (Basel).

[9] Ozcelik N., Ozcelik A.E., Bulbul Y., Oztuna F., Ozlu T. (2020). Can artificial intelligence distinguish between malignant and benign mediastinal lymph nodes using sonographic features on EBUS images?. Curr Med Res Opin.

[10] Moreira I.C., Amaral I., Domingues I., Cardoso A., Cardoso M.J., Cardoso J.S. (2012). INbreast: toward a full-field digital mammographic database. Acad Radiol.

[11] Huang G., Liu Z., Van Der Maaten L., Weinberger K.Q. Densely connected convolutional networks. In: Proceedings of the IEEE conference on computer vision and pattern recognition; 2017. p. 4700–8.

[12] He K., Zhang X., Ren S., Sun J. Deep residual learning for image recognition. In: Proceedings of the IEEE conference on computer vision and pattern recognition; 2016. p. 770–8.

[13] Rajpurkar P. (2017). Chexnet: radiologist-level pneumonia detection on chest x-rays with deep learning. arXiv Prepr.

[14] Krizhevsky A., Sutskever I., Hinton G.E. (2012). Imagenet classification with deep convolutional neural networks. Adv Neural Inf Process Syst.

[15] Atasever S., Azginoglu N., Terzi D.S., Terzi R. (2023). A comprehensive survey of deep learning research on medical image analysis with focus on transfer learning. Clin Imaging.

[16] Anantham D., Koh M.S., Ernst A. (2009). Endobronchial ultrasound. Respir Med.

[17] Zhao Z., Alzubaidi L., Zhang J., Duan Y., Gu Y. (2024). A comparison review of transfer learning and self-supervised learning: definitions, applications, advantages and limitations. Expert Syst Appl.

[18] Wang P., Qiao J., Liu N. (2022). An improved convolutional neural network-based scene image recognition method. Comput Intell Neurosci.

[19] Deng J., Dong W., Socher R., Li L-J, Li K., Fei-Fei L. ImageNet: A large-scale hierarchical image database. 2009 IEEE Conference on Computer Vision and Pattern Recognition, Miami, FL, USA, 2009, pp. 248–255, doi: 10.1109/CVPR.2009.5206848.

[20] Simonyan K., Zisserman A. Very deep convolutional networks for large-scale image recognition. 3rd International Conference on Learning Representations. ICLR 2015 - Conference Track Proceedings. 2014. https://arxiv.org/abs/1409.1556v6.

[21] He K., Zhang X., Ren S., Sun J. (2016). Identity mappings in deep residual networks. Lecture Notes in Computer Science (Including Subseries Lecture Notes in Artificial Intelligence and Lecture Notes in Bioinformatics), 9908 LNCS.

[22] Szegedy C., Liu W., Jia Y., Sermanet P., Reed S., Anguelov D., et al. (2015). Going deeper with convolutions. Proceedings of the IEEE Computer Society Conference on Computer Vision and Pattern Recognition, 07-12-June-2015, 1–9. 10.1109/CVPR.2015.7298594.

[23] Chollet F. (2016). Xception: deep learning with depthwise separable convolutions. 2017 IEEE Conference on Computer Vision and Pattern Recognition (CVPR), Honolulu, HI, USA, 2017, pp. 1800–1807, doi: 10.1109/CVPR.2017.195.

[24] Howard A.G., Zhu M., Chen B., Kalenichenko D., Wang W., Weyand T., et al. MobileNets: efficient convolutional neural networks for mobile vision applications. 2017. https://arxiv.org/abs/1704.04861v1.

[25] Huang G., Liu Z., Van Der Maaten L., Weinberger K.Q. Densely connected convolutional networks. 2017 IEEE Conference on Computer Vision and Pattern Recognition (CVPR), Honolulu, HI, USA, 2017, pp. 2261–2269, doi: 10.1109/CVPR.2017.243.

[26] Zoph B., Vasudevan V., Shlens J., Le Q.V. (2017). Proceedings of the IEEE Computer Society Conference on Computer Vision and Pattern Recognition.

[27] Tan M., Le Q.V. (2021). EfficientNetV2: smaller models and faster training. Proceed Mach Learn Res.

[28] Li C., Yan Y., Xu H., Cao H., Zhang J., Sha J. (2022). Comparison of transfer learning models in pelvic tilt and rotation measurement in pediatric anteroposterior pelvic radiographs. J Digit Imaging.

[29] Ayana H., Sileshi T., Bule M.H., Chaka E.E. (2021). Non-prescription antibiotics use and associated factors among drug retail outlets in Ambo, Ethiopia: a cross-sectional study. Patient Prefer Adherence.

[30] Masud M., Rashed A., Hossain M. (2020). Convolutional neural network-based models for diagnosis of breast cancer. Neural Comput Appl.

[31] Cheng P., Malhi H. (2016). Transfer learning with convolutional neural networks for classification of abdominal ultrasound images. J Digit Imaging.

[32] Saha S., Sheikh N. (2021). Ultrasound image classification using acgan with small training dataset. Annu Int Conf IEEE Eng Med Biol Soc.

[33] Wu X., Li M., Cui X., Xu G. (2022). Deep multimodal learning for lymph node metastasis prediction of primary thyroid cancer. Phys Med Biol.

[34] Chen W., Gu Z., Liu Z., Fu Y., Ye Z., Zhang X. (2021). A new classification method in ultrasound images of benign and malignant thyroid nodules based on transfer learning and deep convolutional neural network. Complex.

[35] Navani N., Nankivell M., Lawrence D., Löck S., Makker H., Baldwin D. (2015). Lung-BOOST trial investigators. Lung cancer diagnosis and staging with endobronchial ultrasound-guided transbronchial needle aspiration compared with conventional approaches: an open-label, pragmatic, randomised controlled trial. Lancet Respir Med.

[36] Ervik Ø., Rødde M., Hofstad E.F., Tveten I., Langø T., Leira H.O. (2025). A new deep learning-based method for automated identification of thoracic lymph node stations in endobronchial ultrasound (EBUS): a proof-of-concept study. J Imaging.

[37] Yang X., Chen Z., Jia X. (2022). Deep learning algorithm-based ultrasound image information in diagnosis and treatment of pernicious placenta previa. Comput Math Methods Med.

[38] Koseoglu F.D., Alıcı I.O., Er O. (2023). Machine learning approaches in the interpretation of endobronchial ultrasound images: a comparative analysis. Surg Endosc.

[39] Patel Y.S., Gatti A.A., Farrokhyar F., Xie F., Hanna W.C. (2024). Artificial intelligence algorithm can predict lymph node malignancy from endobronchial ultrasound transbronchial needle aspiration images for non-small cell lung cancer. Respiration.

[40] Ito Y., Nakajima T., Inage T., Otsuka T., Sata Y., Tanaka K. (2022). Prediction of nodal metastasis in lung cancer using deep learning of endobronchial ultrasound images. Cancers (Basel).

